# Differences in Regional Patterns of Influenza Activity Across Surveillance Systems in the United States: Comparative Evaluation

**DOI:** 10.2196/13403

**Published:** 2019-09-14

**Authors:** Kristin Baltrusaitis, Alessandro Vespignani, Roni Rosenfeld, Josh Gray, Dorrie Raymond, Mauricio Santillana

**Affiliations:** 1 Department of Biostatistics, Boston University School of Public Health Boston, MA United States; 2 Institute for Network Science, Northeastern University Boston, MA United States; 3 School of Computer Science, Carnegie Mellon University Pittsburgh, PA United States; 4 athenaResearch at athenahealth Watertown, MA United States; 5 Computational Health Informatics Program Boston Children’s Hospital Boston, MA United States; 6 Department of Pediatrics, Harvard Medical School Boston, MA United States

**Keywords:** digital disease surveillance, influenza, surveillance, participatory syndromic surveillance, disease modeling

## Abstract

**Background:**

The Centers for Disease Control and Prevention (CDC) tracks influenza-like illness (ILI) using information on patient visits to health care providers through the Outpatient Influenza-like Illness Surveillance Network (ILINet). As participation in this system is voluntary, the composition, coverage, and consistency of health care reports vary from state to state, leading to different measures of ILI activity between regions. The degree to which these measures reflect actual differences in influenza activity or systematic differences in the methods used to collect and aggregate the data is unclear.

**Objective:**

The objective of our study was to qualitatively and quantitatively compare national and region-specific ILI activity in the United States across 4 surveillance data sources—CDC ILINet, Flu Near You (FNY), athenahealth, and HealthTweets.org—to determine whether these data sources, commonly used as input in influenza modeling efforts, show geographical patterns that are similar to those observed in CDC ILINet’s data. We also compared the yearly percentage of FNY participants who sought health care for ILI symptoms across geographical areas.

**Methods:**

We compared the national and regional 2018-2019 ILI activity baselines, calculated using noninfluenza weeks from previous years, for each surveillance data source. We also compared measures of ILI activity across geographical areas during 3 influenza seasons, 2015-2016, 2016-2017, and 2017-2018. Geographical differences in weekly ILI activity within each data source were also assessed using relative mean differences and time series heatmaps. National and regional age-adjusted health care–seeking percentages were calculated for each influenza season by dividing the number of FNY participants who sought medical care for ILI symptoms by the total number of ILI reports within an influenza season. Pearson correlations were used to assess the association between the health care–seeking percentages and baselines for each surveillance data source.

**Results:**

We observed consistent differences in ILI activity across geographical areas for CDC ILINet and athenahealth data. ILI activity for FNY displayed little variation across geographical areas, whereas differences in ILI activity for HealthTweets.org were associated with the total number of tweets within a geographical area. The percentage of FNY participants who sought health care for ILI symptoms differed slightly across geographical areas, and these percentages were positively correlated with CDC ILINet and athenahealth baselines.

**Conclusions:**

Our findings suggest that differences in ILI activity across geographical areas as reported by a given surveillance system may not accurately reflect true differences in the prevalence of ILI. Instead, these differences may reflect systematic collection and aggregation biases that are particular to each system and consistent across influenza seasons. These findings are potentially relevant in the real-time analysis of the influenza season and in the definition of unbiased forecast models.

## Introduction

### Background

Influenza epidemics are responsible for a significant public health burden that includes an estimated 12,000 to 56,000 deaths each year in the United States [1]. Consequently, timely and reliable surveillance of influenza activity is essential for local, state, and national public health officials to monitor and respond to outbreaks. In the United States, the Centers for Disease Control and Prevention (CDC) collects and analyzes information on influenza activity throughout the year. As part of this national surveillance system, patients seeking medical attention for influenza-like illness (ILI) are tracked through the Outpatient Influenza-like Illness Surveillance Network (ILINet). This system contains thousands of volunteer health care specialists, including individual providers, group practices, and hospital-based clinics located throughout all 50 states, Puerto Rico, the District of Columbia, and the US Virgin Islands. As participation in ILINet is voluntary and each state is responsible for their own recruitment of health care providers, the composition of provider types, coverage of geographical regions, and consistency of provider reporting vary from state to state. This convenience sample–driven model of surveillance results in certain parts of the population being over- or underrepresented in the reported influenza activity [2-4].

At both national and Health and Human Services–defined regional levels (conglomerates of 2-8 states), the CDC routinely reports the weekly percentage of patients presenting with ILI to health care providers. In addition, the CDC calculates and reports region-specific baselines *,* using influenza activity data from previous seasons, to identify the beginning and end of the influenza season and contextualize the severity of a given region-specific outbreak. These baselines vary widely across regions, and the degree to which the differences in baselines, as well as the percentage of ILI visits during an influenza season, reflect actual differences in influenza activity or systematic differences in the methods used to collect the data is unclear. Recent models suggest that the spatial patterns in US sentinel ILI surveillance may be the result of socioenvironmental factors, state-specific health policies, and sampling [3]. Identifying and characterizing the presence of potential methodological measurement biases in ILINet is important, as it is frequently used as an indicator of influenza activity for decision-making purposes and as the ground truth in mechanistic and statistical predictive modeling efforts aimed at understanding disease transmission dynamics and monitoring and forecasting influenza activity [5-15]. Furthermore, because these models typically leverage data from outside of the public health systems, such as Google internet searches [15,16], participatory syndromic surveillance systems [17,18], Twitter [19], and electronic health record (EHR) [14,20], it is important to understand if input sources show similar structural aggregation patterns.

### Objectives

In this study, we qualitatively and quantitatively compared national and region-specific baselines and ILI activity during 3 influenza seasons across 4 surveillance data sources—CDC ILINet; Flu Near You (FNY), a crowd-sourced participatory syndromic surveillance system; athenahealth, a provider of cloud-based EHR services; and HealthTweets.org, a research platform that shares health trends data from Twitter—to determine whether these surveillance data sources, commonly used as input in influenza modeling efforts, show regional structural patterns that are similar to those observed in CDC ILINet’s data. We also compared yearly self-reported health care–seeking rates of FNY participants to determine if this factor can better characterize the differences in ILI activity across geographic areas.

## Methods

### Data

#### Centers for Disease Control and Prevention Outpatient Influenza-Like Illness Surveillance Network

The CDC reports the weighted percentage of patient visits to health care providers presenting ILI symptoms on a weekly basis at the national and regional levels. These values are weighted on the basis of state population and represent the percentage of patient visits to health care providers that present as ILI, defined as fever (temperature of 100°F [37.8°C] or greater) plus a cough and/or a sore throat without a known cause other than influenza.

#### Flu Near You

FNY is a participatory syndromic surveillance system that allows volunteers in the United States to report health information of the user and their family using brief weekly surveys [21]. Through these surveys, FNY users report any symptoms that they or any registered household members experienced during the previous week (Monday through Sunday). For all reported symptoms, FNY users are asked to provide the date of symptom(s) onset and whether or not they received medical care for the symptom(s). The national and regional percentage of ILI symptoms reported is calculated by dividing the number of participants reporting ILI, as defined by reporting fever plus cough and/or sore throat, in a given week by the total number of FNY participant reports in that same week. FNY participants are assigned to a region based on the zip code provided at registration. Unweighted FNY percentage of ILI symptoms is used to maintain consistency across previous studies and the FNY website.

athenahealth is a provider of cloud-based services and mobile apps for medical groups and health systems. National and regional percentage of visits for ILI is calculated by dividing the unspecified viral or ILI visit count, which is equal to the number of visits where the patient had an unspecified viral diagnosis, an influenza diagnosis, or a fever diagnosis with an accompanying sore throat or cough diagnosis, by the total number of visits for each week.

#### HealthTweets.org

This dataset is generated by a Web-based research platform (HealthTweets.org) that shares the output of Twitter data mining algorithms with researchers and public officials [19]. We use weekly aggregated trends data from each state to calculate the influenza prevalence measure for each region. Weekly national and regional influenza prevalence measures are calculated by normalizing the number of influenza infection tweets in the health stream by the total number of tweets in the general stream during the same week [22].

### Statistics of Datasets

#### Baseline Comparison

Baselines are used as a single quantitative measure that compares ILI activity during noninfluenza weeks across geographical areas within each surveillance data source. The CDC ILINet national and regional baselines for the 2018-2019 influenza season are available on the CDC website [23]. National and regional baselines for FNY, athenahealth, and HealthTweets.org are estimated following the CDC’s baseline definition. A baseline is defined as the mean percentage of ILI activity during *noninfluenza weeks,* for the previous 3 seasons, plus 2 SDs. *Noninfluenza weeks* during these seasons are the same for all 3 systems and are delineated, by the CDC, as periods of 2 or more consecutive weeks in which each week accounted for less than 2% of the season’s total number of specimens that tested positive for influenza in public health laboratories. We used region-specific noninfluenza weeks. Descriptive statistics of baselines are presented as median (interquartile range, IQR).

#### Influenza-Like Illness Activity Comparison

**Figure 1:**
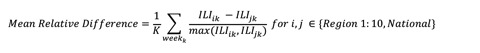
Equation for the mean relative difference.

Differences in ILI activity during noninfluenza as well as influenza weeks across geographical areas within each surveillance data source are assessed using data from the start of the 2015-2016 influenza season (week of October 5, 2015) to the end of the 2017-2018 influenza season (week of October 1, 2018). Weekly ILI activity across geographical areas within each data source is quantitatively compared by dividing the difference in ILI activity between 2 areas by the maximum within each week, defined by Figure 1.

Mean relative differences within each surveillance data source are summarized using heatmaps, where the geographical areas along the rows are represented by *i* in the equation and the geographical areas along the columns are represented by *j*. Geographical areas that have consistently higher weekly ILI activity compared with other geographical areas have positive mean relative differences, indicated by red shades across the row in the heatmap, whereas geographical areas that have consistently lower weekly ILI activity have negative mean relative differences, indicated by blue shades across the row. Time series heatmaps are also presented to qualitatively compare weekly ILI activity across geographical areas for each surveillance data source.

#### Health Care–Seeking Behavior

National and regional health care–seeking percentages are calculated for each influenza season by dividing the number of FNY participants who sought medical care for ILI symptoms, as defined above, by the total number of ILI reports within an influenza season. As health care–seeking behavior varies by age [24], health care–seeking percentages are adjusted by age group (<18 years, 18-49 years, 50-64 years, and ≥65 years) using population data from the 2010 US census [25]. We use Pearson correlation to assess the association between the adjusted health care–seeking percentages and baselines for each surveillance data source. All analyses are performed using R version 3.3.2. [26].

## Results

### Centers for Disease Control and Prevention Outpatient Influenza-Like Illness Surveillance Network

**Figure 2:**
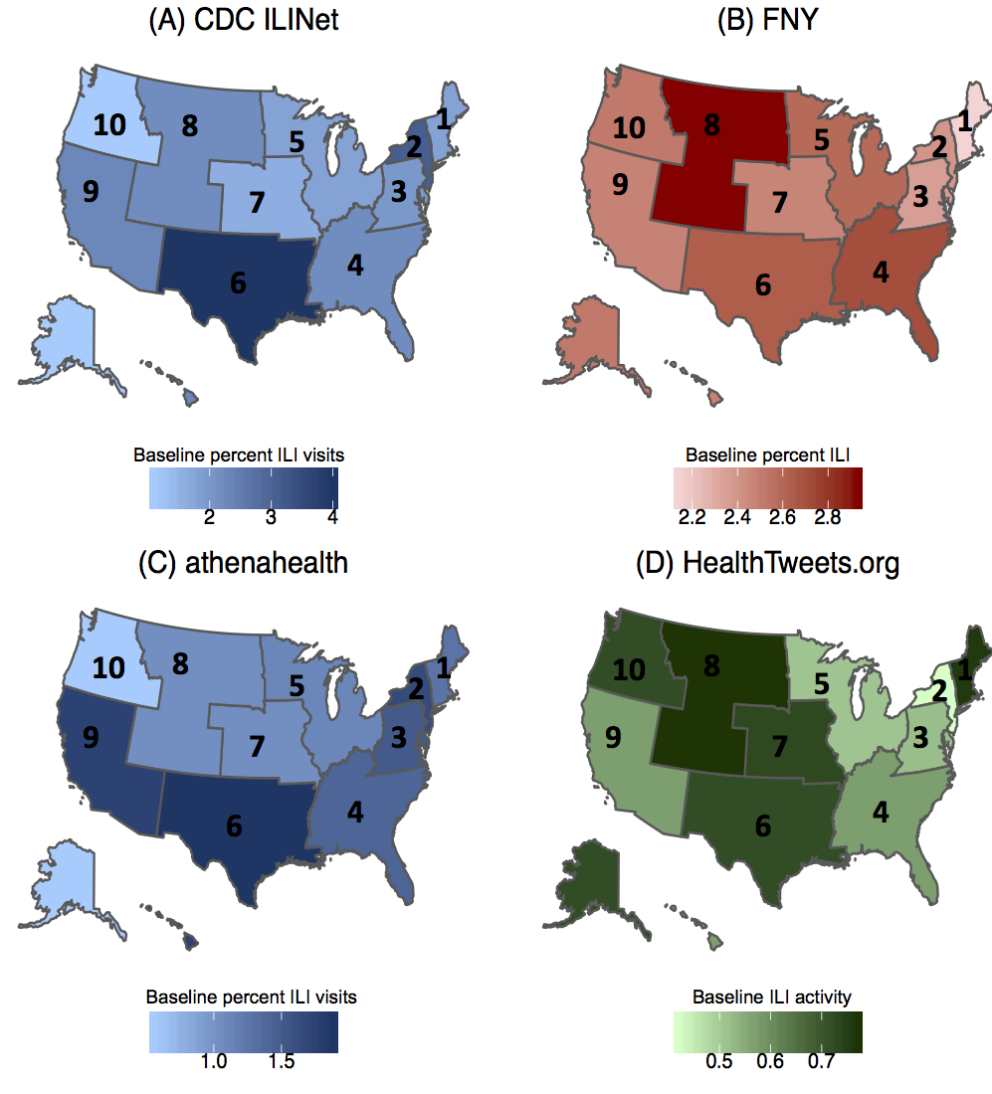
Spatial heatmaps of US regional baseline influenza-like illness activity for the 2018-2019 influenza season for (A) Centers for Disease Control and Prevention Influenza-like Illness Surveillance Network, (B) Flu Near You, (C) athenahealth, and (D) HealthTweets.org. CDC: Centers for Disease Control and Prevention; FNY: Flu Near You; ILINet: Influenza-like Illness Surveillance Network.

**Figure 3:**
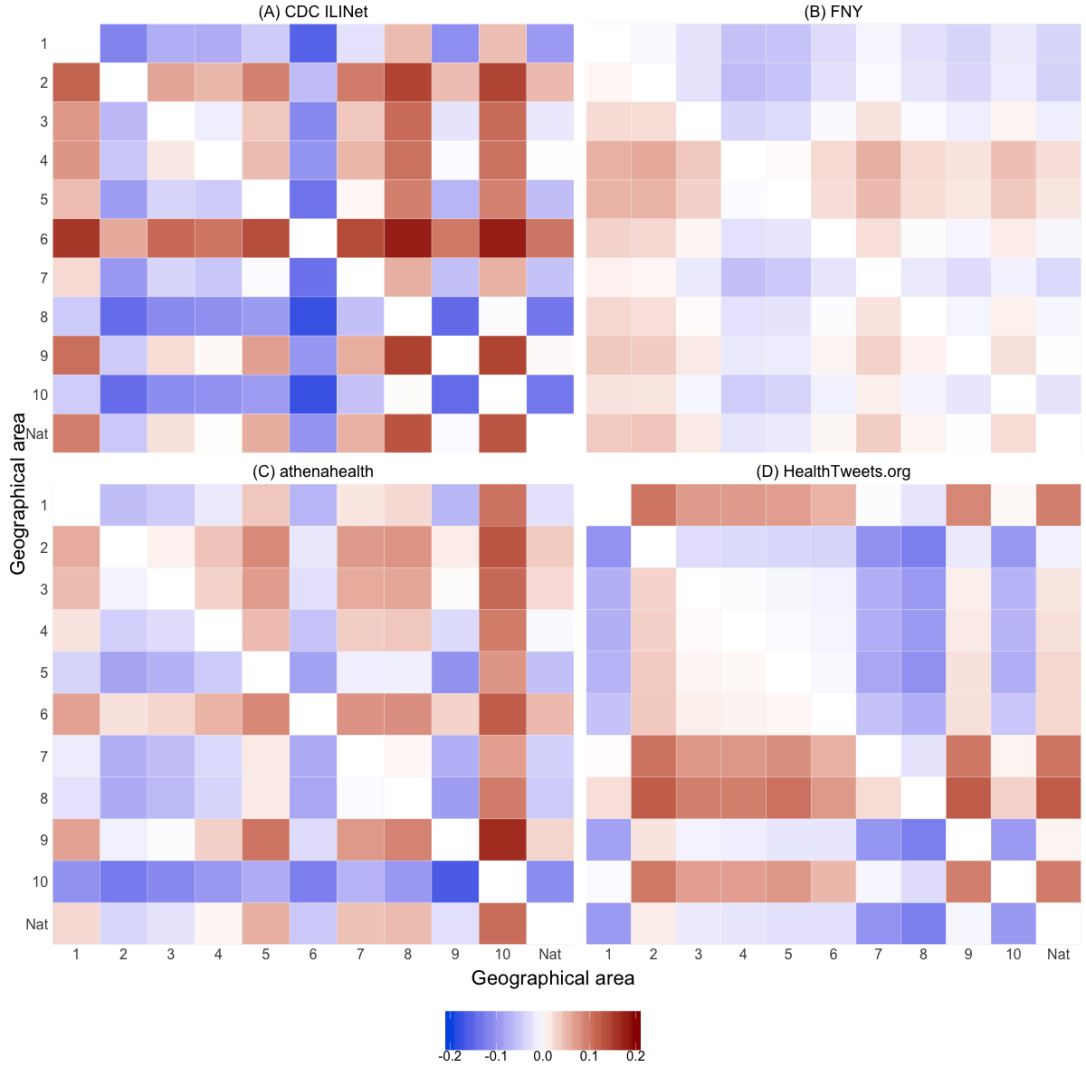
Heatmaps of the mean relative difference of influenza-like illness activity across geographical areas for (A) Centers for Disease Control and Prevention Influenza-like Illness Surveillance Network, (B) Flu Near You, (C) athenahealth, and (D) HealthTweets.org. CDC: Centers for Disease Control and Prevention; FNY: Flu Near You; ILINet: Influenza-like Illness Surveillance Network.

**Figure 4:**
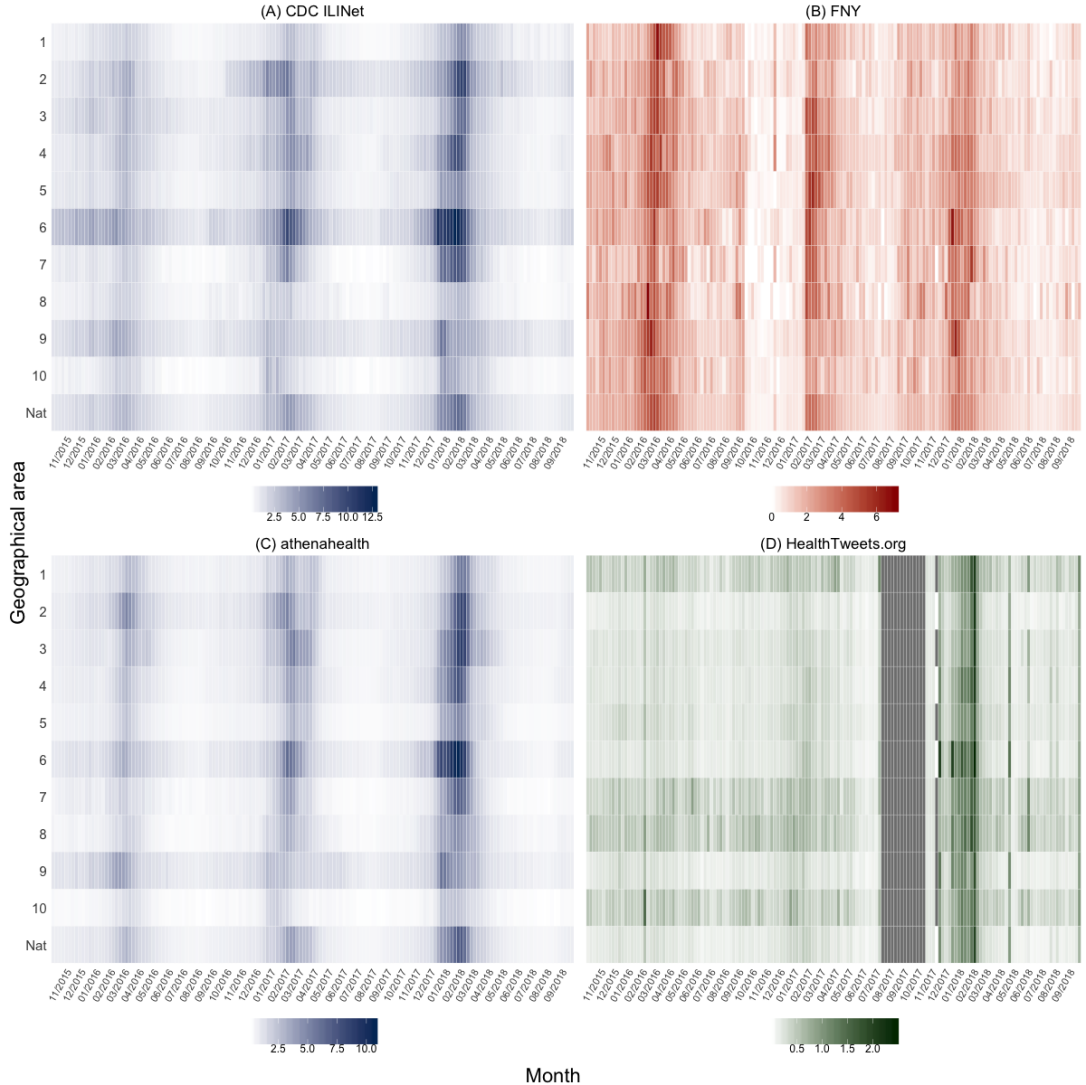
Time series heatmaps of influenza-like illness activity across geographical areas for (A) Centers for Disease Control and Prevention Influenza-like Illness Surveillance Network, (B) Flu Near You, (C) athenahealth, and (D) HealthTweets.org. CDC: Centers for Disease Control and Prevention; FNY: Flu Near You; ILI: influenza-like illness ILINet: Influenza-like Illness Surveillance Network.

Table 1 and Figure 2 provide the ILI activity baselines for each surveillance data source across geographical areas. The national baseline for CDC ILINet during the 2018-2019 influenza season is 2.2, and the median CDC ILINet regional baseline is 2.1 (IQR 1.8-2.3). Region 10 has the smallest baseline, 1.1, whereas region 6 has the largest baseline, 4.0. As shown in Figure 3 regions 2 and 6 have consistently higher weekly percentage of ILI visits compared with other regions, indicated by the red shades across the row, whereas regions 1, 8, and 10 have consistently lower weekly percentage of ILI visits, indicated by the blue shades across the row. This pattern is also shown qualitatively in both Figure 4 and Multimedia Appendix 1, where darker shades of red, as seen for regions 2, 6, and 9, correspond to higher percentage of ILI visits.

**Table 1:** Regional and national influenza-like illness activity baselines for the 2018-2019 influenza season for Centers for Disease Control and Prevention Outpatient Influenza-like Illness Surveillance Network, Flu Near You, athenahealth, and HealthTweets.org.

Geographical area	Centers for Disease Control and Prevention Outpatient Influenza-like Illness Surveillance Network	Flu Near You	athenahealth	HealthTweets.org
Region 1^a^	1.8	2.1	1.3	0.8
Region 2^b^	3.1	2.4	1.7	0.4
Region 3^c^	2.0	2.4	1.5	0.5
Region 4^d^	2.2	2.7	1.4	0.6
Region 5^e^	1.8	2.6	1.1	0.5
Region 6^f^	4.0	2.6	1.9	0.7
Region 7^g^	1.6	2.5	1.0	0.7
Region 8^h^	2.2	2.9	1.0	0.8
Region 9^i^	2.3	2.5	1.7	0.6
Region 10^j^	1.1	2.5	0.6	0.7
National	2.2	2.3	1.4	0.5

^a^Region 1 includes Connecticut, Maine, Massachusetts, New Hampshire, Rhode Island, and Vermont.

^b^Region 2 includes New Jersey, New York, Puerto Rico, and US Virgin Islands.

^c^Region 3 includes Delaware, District of Columbia, Maryland, Pennsylvania, Virginia, and West Virginia.

^d^Region 4 includes Alabama, Florida, Georgia, Kentucky, Mississippi, North Carolina, South Carolina, and Tennessee.

^e^Region 5 includes Illinois, Indiana, Michigan, Minnesota, Ohio, and Wisconsin.

^f^Region 6 includes Arkansas, Louisiana, New Mexico, Oklahoma, and Texas.

^g^Region 7 includes Iowa, Kansas, Missouri, and Nebraska.

^h^Region 8 includes Colorado, Montana, North Dakota, South Dakota, Utah, and Wyoming.

^i^Region 9 includes Arizona, California, Guam, Hawaii, and Nevada.

^j^Region 10 includes Alaska, Idaho, Oregon, and Washington.

### Flu Near You

For FNY, the national baseline is 2.3, and the median regional baseline is 2.5 (IQR 2.4-2.6). The minimum baseline is 2.1, region 1, and the maximum baseline is 2.9, region 8. Compared with other data sources, the mean relative differences for FNY in Figure 3 show less heterogeneity and no consistent patterns in the percentage of ILI across geographical areas. Although the timing of peaks in the percentage of ILI varies between regions, the relative percentage of ILI is consistent across geographical areas and seasons (Figure 4 and Multimedia Appendix 2).

The national baseline for athenahealth is 1.4, and the median regional baseline is 1.3 (IQR 1.0-1.6). Region 10 has the minimum baseline of 0.6, and region 6 has the maximum baseline of 1.9. Similar to CDC ILINet, regions 2, 6, and 9 have consistently higher weekly percentage of ILI visits compared with other regions, and regions 7, 8, and 10 have consistently lower weekly percentage of ILI visits. This pattern is reflected in Figure 4 and Multimedia Appendix 3, as regions 2, 6, and 9 have consistently higher percentage of ILI visits across all seasons.

### HealthTweets.org

The national baseline is 0.5, the median baseline is 0.6 (IQR 0.5-0.7), the minimum baseline is 0.4 (region 2), and the maximum baseline is 0.8 (region 8). Unlike CDC ILINet and athenahealth, HealthTweets.org shows higher ILI activity in regions 1, 7, 8, and 10 (Figure 3). These regions have mean normalizing constants that are less than half the mean normalizing constants of other regions (Table 2). As shown in Figure 4 and Multimedia Appendix 4, this pattern is consistent across seasons.

**Table 2:** Descriptive statistics of the HealthTweets.org normalizing constant at the national and regional levels.

Geographical area	Normalizing constant, mean (SD)
Region 1^a^	210.82 (114.917)
Region 2^b^	627.69 (330.270)
Region 3^c^	599.53 (293.320)
Region 4^d^	1103.78 (553.374)
Region 5^e^	798.25 (387.266)
Region 6^f^	845.30 (414.785)
Region 7^g^	171.05 (82.077)
Region 8^h^	121.96 (63.936)
Region 9^i^	5848.54 (3775.923)
Region 10^j^	181.33 (97.756)
National	6352.25 (3351.390)

^a^Region 1 includes Connecticut, Maine, Massachusetts, New Hampshire, Rhode Island, and Vermont.

^b^Region 2 includes New Jersey, New York, Puerto Rico, and US Virgin Islands.

^c^Region 3 includes Delaware, District of Columbia, Maryland, Pennsylvania, Virginia, and West Virginia.

^d^Region 4 includes Alabama, Florida, Georgia, Kentucky, Mississippi, North Carolina, South Carolina, and Tennessee.

^e^Region 5 includes Illinois, Indiana, Michigan, Minnesota, Ohio, and Wisconsin.

^f^Region 6 includes Arkansas, Louisiana, New Mexico, Oklahoma, and Texas.

^g^Region 7 includes Iowa, Kansas, Missouri, and Nebraska.

^h^Region 8 includes Colorado, Montana, North Dakota, South Dakota, Utah, and Wyoming.

^i^Region 9 includes Arizona, California, Guam, Hawaii, and Nevada.

^j^Region 10 includes Alaska, Idaho, Oregon, and Washington.

### Health Care–Seeking Behavior

**Figure 5:**
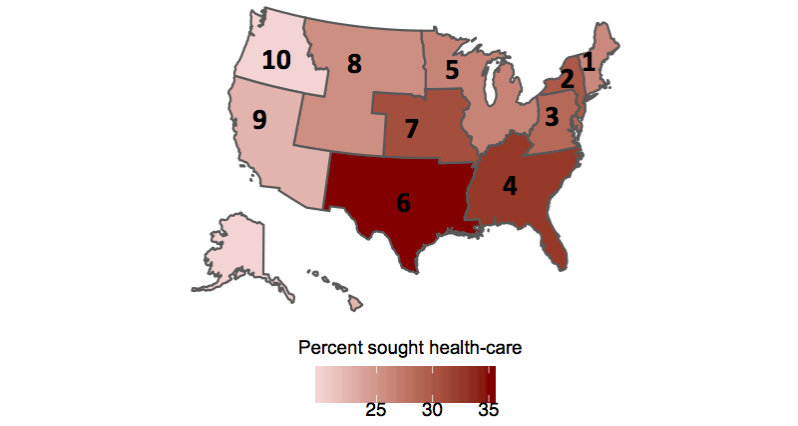
Spatial heatmap of age-adjusted regional percentage of Flu Near You participants who sought health care for ILI symptoms across all seasons.

The age-adjusted estimates of the percentage of FNY participants who sought health care for ILI symptoms are shown by season and across all seasons in Table 3 and Figure 5. At the national level, a higher age-adjusted percentage of participants sought health care for ILI symptoms during the 2016-2017 season, 35.1%, compared with the 2015-2016 and 2017-2018 seasons, 21.7% and 29.2%, respectively. Within each season, regions 2, 4, and 6 have the highest age-adjusted percentages of participants who sought health care, whereas regions 1, 5, 9, and 10 have the smallest age-adjusted percentages of participants who sought health care.

**Figure 6:**
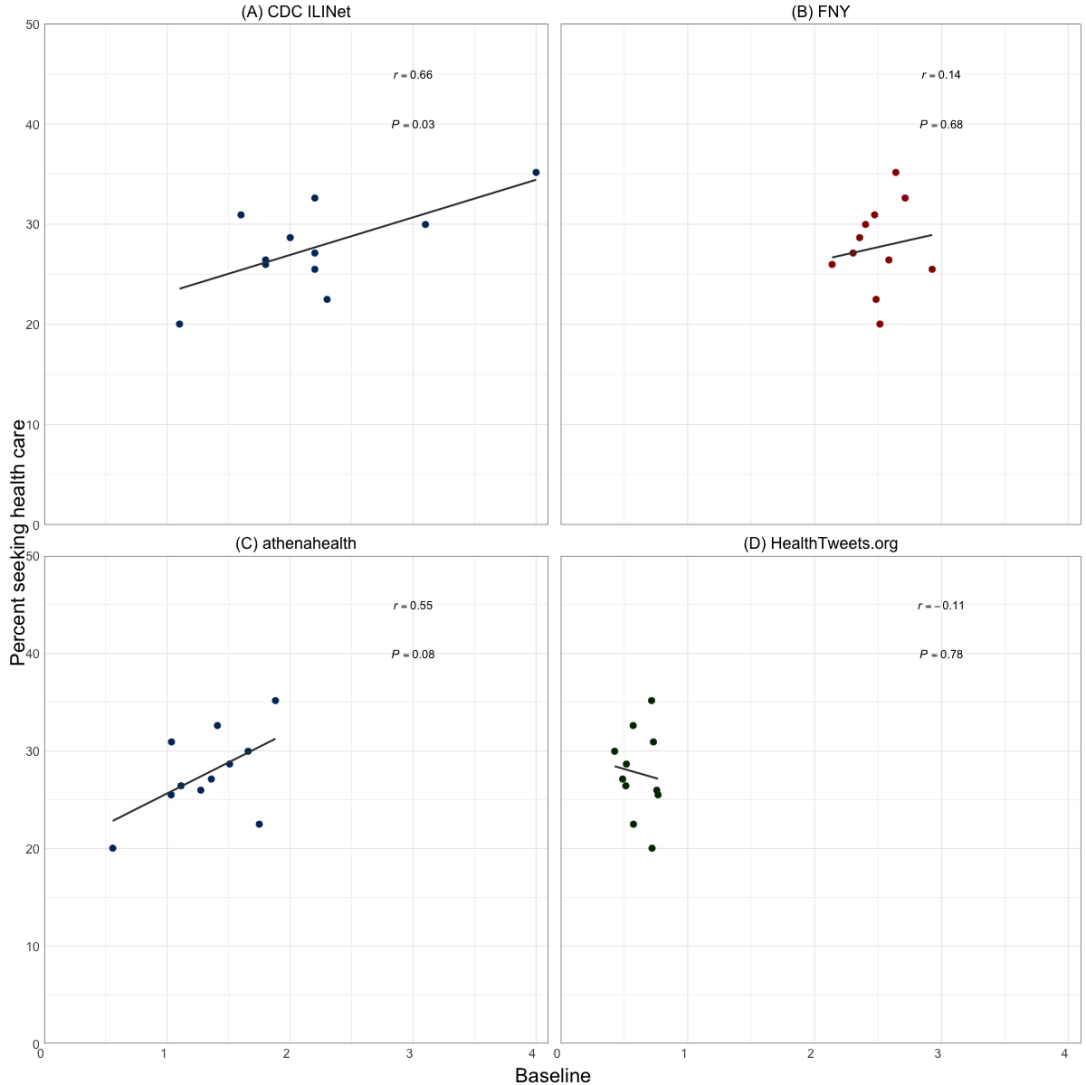
Scatterplot of the age-adjusted percentage of individuals who sought health care for influenza-like illness symptoms versus baseline influenza-like illness activity for (A) Centers for Disease Control and Prevention Influenza-like Illness Surveillance Network, (B) Flu Near You, (C) athenahealth, and (D) HealthTweets.org. CDC: Centers for Disease Control and Prevention; FNY: Flu Near You; ILINet: Influenza-like Illness Surveillance Network.

As shown in Figure 6, the age-adjusted estimates of the percentage of individuals who sought health care for ILI symptoms is significantly correlated with the baselines for CDC ILINet (*P*=.03) and is borderline significant for athenahealth (*P*=.08). There is no evidence of an association between the age-adjusted estimates of the percentage of individuals who sought health care and the baselines for FNY (*P*=.68) and HealthTweets.org (*P*=.76).

**Table 3:** Age-adjusted regional and national estimates of the percentage of Flu Near You participants who sought health care for influenza-like illness symptoms.

Geographical area	All seasons	2015-2016	2016-2017	2017-2018
Region 1^a^	25.98	20.82	33.29	27.77
Region 2^b^	29.97	26.05	36.03	31.79
Region 3^c^	28.66	22.07	37.03	31.73
Region 4^d^	32.61	25.47	43.23	34.77
Region 5^e^	26.43	21.53	34.59	26.73
Region 6^f^	35.17	28.58	44.83	37.47
Region 7^g^	30.93	23.79	41.95	32.09
Region 8^h^	25.50	22.74	30.86	26.16
Region 9^i^	22.49	19.06	27.77	24.69
Region 10^j^	20.03	17.03	23.39	22.33
National	27.12	21.73	35.06	29.23

^a^Region 1 includes Connecticut, Maine, Massachusetts, New Hampshire, Rhode Island, and Vermont.

^b^Region 2 includes New Jersey, New York, Puerto Rico, and US Virgin Islands.

^c^Region 3 includes Delaware, District of Columbia, Maryland, Pennsylvania, Virginia, and West Virginia.

^d^Region 4 includes Alabama, Florida, Georgia, Kentucky, Mississippi, North Carolina, South Carolina, and Tennessee.

^e^Region 5 includes Illinois, Indiana, Michigan, Minnesota, Ohio, and Wisconsin.

^f^Region 6 includes Arkansas, Louisiana, New Mexico, Oklahoma, and Texas.

^g^Region 7 includes Iowa, Kansas, Missouri, and Nebraska.

^h^Region 8 includes Colorado, Montana, North Dakota, South Dakota, Utah, and Wyoming.

^i^Region 9 includes Arizona, California, Guam, Hawaii, and Nevada.

^j^Region 10 includes Alaska, Idaho, Oregon, and Washington.

## Discussion

### Principal Findings

Our findings show that differences in ILI activity across regions, as reported by a given surveillance system, are not consistent across surveillance platforms. In other words, regions that show larger baselines (and thus higher overall historical ILI activity) in one surveillance system appear to be different from their counterparts in other surveillance systems. The heterogeneity of recruitment practices of health care providers for each system, the composition of provider types, and the variability and consistency of coverage of geographical regions have the potential to contribute substantially to these systematic differences in baselines [3]. As such, our findings suggest that these structural differences reflect methodological collection practices rather than actual differences in influenza activity across regions. The observed structural patterns within each surveillance system are consistent across individual influenza seasons (Multimedia Appendix 5), which implies that the differences are not reflecting a specific time-period heterogeneity.

Specifically, baselines from CDC ILINet vary across different geographical areas, and the geographical areas with the largest baselines also have a consistently larger percentage of ILI visits during the influenza season. Conversely, FNY’s baselines and the percentage of ILI were similar across geographical areas. This similarity is captured by the homogeneity in the mean relative differences. One potential contributing factor to the observed differences in patterns between these surveillance systems is the activity being measured. CDC ILINet measures the number of visits with ILI out of the total number of patient visits, whereas FNY measures the number of ILI reports out of enrolled persons who submitted a report. Furthermore, the population under surveillance also differs as FNY includes individuals who may not seek medical attention and FNY has a different demographic profile compared with CDC ILINet. For example, females and middle-aged participants are overrepresented in FNY [27].

Although not identical, athenahealth shows similar patterns in both baseline measures as well as the percentage of ILI visits to CDC ILINet across geographical areas. Both CDC ILINet and athenahealth use data from individuals seeking medical care. However, athenahealth has only a partially overlapping coverage of health care providers, and the proportion of visit settings differs slightly between the 2 systems. Most of athenahealth’s providers see patients in office-based settings. Other settings, such as emergency room and nursing facilities, are underrepresented compared with CDC ILINet [28].

Unlike FNY, patterns across geographical areas within HealthTweets.org ILI activity appear to be the opposite of the patterns shown by CDC ILINet and athenahealth, as areas with consistently lower HealthTweets.org ILI activity have a consistently higher percentage of ILI visits for CDC ILINet and athenahealth, and vice versa. One potential reason for the differences in patterns in ILI activity across data sources is the difference in the activity being measured. As mentioned above, both CDC ILINet and athenahealth measure the number of ILI visits out of total visits, whereas HealthTweets.org normalizes the number of influenza infection tweets by the total number of tweets in the general stream. In addition, the groups most susceptible to influenza illness, young children and the elderly, may be underrepresented on Twitter. Furthermore, we found that smaller normalizing constants correspond to higher values of ILI activity.

### Comparison With Previous Work

Despite the differences in patterns of ILI activity within systems, current research shows that these alternative data sources track CDC ILINet at both the national and regional levels. At the national level, the correlation between CDC ILINet and athenahealth is 0.97, and regional correlations range from 0.90 to 0.97 [29]. The correlation between CDC ILINet and FNY at the national level is 0.81, and regional correlations range from 0.64 to 0.81 [29]. Twitter-based influenza prevalence measures show a correlation of 0.93 with CDC ILINet at the national level and a correlation of 0.88 with New York City’s weekly emergency department visits for ILI [22].

Compared with other recent publications, the percentage of FNY participants who sought medical care for ILI is less than reported estimates. A recent meta-analysis that used estimates from multiple countries across different influenza seasons estimated an overall pooled health care–seeking rate of 0.52 (95% CI 0.46-0.59) [30]. In the United States, national reported health care–seeking percentages for children were 56% and 57% during the 2009-2010 and 2010-2011 influenza seasons, respectively. Among adults, 40% reported that they sought health care during the 2009-2010 influenza season and 45% reported that they sought health care during the 2009-2010 influenza season [24,31]. Interestingly, the percentage of FNY participants who sought health care for ILI symptoms differs slightly across geographical areas. These differences may contribute to the differences in CDC ILINet and athenahealth baseline activity, as health care–seeking percentages are positively correlated with both CDC ILINet and athenahealth baselines.

From a predictive modeling perspective, our findings may explain why certain approaches designed to predict CDC ILINet values for the *Predict the Influenza season challenge*, weeks ahead of the publication of official CDC reports, may work better than others. As discussed in the 2 existing reports that document the performance of different methodologies to predict influenza activity, models that rely on local statistical approaches that exploit region-specific autoregressive information and historically observed ILI activity from previous seasons, as well as external predictors (such as humidity data, Google searches, and Wikipedia) [9,11], outperform mechanistic agent-based stochastic susceptible-infected-recovered (SIR) models that aim at modeling individual humans’ behavior to infer epidemic activity across spatial resolutions [7,8,10]. The former modeling approaches are *trained* to track ILI levels in a region-specific fashion (frequently ignoring inconsistency across spatial resolutions), whereas the latter agent-based stochastic SIR models aim to predict the whole national epidemic outbreak across geographic areas. In other words, if the ILI activity report varies depending on how data are aggregated, then even a very accurate agent-based model may not be able to capture influenza activity correctly for every geographic area.

### Limitations

Our study has several limitations. During the beginning of the 2015-2016 season, there were errors in FNY data collection, resulting in an underestimation in the weekly percentage of ILI reports. We did not adjust the estimates of these weeks. There was also an issue in data collection during the week of August 28, 2017. We adjusted the estimates for this week by taking the average percentage of ILI reports of the previous and subsequent weeks. In addition, there were a few weeks during the summer of 2017 during which there were no reports of ILI activity for HealthTweets.org. We did not input or estimate these missing weeks. As the overall patterns of ILI activity were similar across seasons (Multimedia Appendix 5), we do not suspect that these data issues affected our overall conclusions.

In addition, FNY relies on self-reported data that are subject to recall and social desirability bias. FNY participant reporting is also not consistent throughout the influenza season. Although previous students have used various methods, including restricting analyses to cohorts of users that report regularly [32-34], dropping the first report of all users, and applying a spike detector [21], we did not adjust for these potential reporting biases because reporting habits are consistent across regions [27]. Finally, because each system has a different measure of ILI activity, we cannot directly compare measures across systems.

### Conclusions

Although ILI activity differs across geographical areas and data sources, the general region-specific seasonal trends are similar and provide valuable information about changes in influenza activity. Together, these platforms offer a more comprehensive view of influenza surveillance that helps public health offices monitor and respond to seasonal influenza epidemics.

## Appendix

Multimedia Appendix 1 Time series plots of weekly percentage of influenza-like illness visits from Centers for Disease Control and Prevention Influenza-like Illness Surveillance Network across 3 influenza seasons (2015-2016, 2016-2017, and 2017-2018) with baselines. Geographical areas on the columns are presented in black, and geographical areas on the rows are presented in blue.

Multimedia Appendix 2 Time series plots of weekly percentage of influenza-like illness from Flu Near You across 3 influenza seasons (2015-2016, 2016-2017, and 2017-2018) with baselines. Geographical areas on the columns are presented in black, and geographical areas on the rows are presented in red.

Multimedia Appendix 3 Time series plots of weekly percentage of influenza-like illness visits from athenahealth across 3 influenza seasons (2015-2016, 2016-2017, and 2017-2018) with baselines. Geographical areas on the columns are presented in black, and geographical areas on the rows are presented in blue.

Multimedia Appendix 4 Time series plots of weekly influenza-like illness activity from HealthTweets.org across 3 influenza seasons (2015-2016, 2016-2017, and 2017-2018) with baselines. Geographical areas on the columns are presented in black, and geographical areas on the rows are presented in green.

Multimedia Appendix 5 Heatmaps of the mean relative difference of ILI activity across geographical areas for (A) Centers for Disease Control and Prevention Influenza-like Illness Surveillance Network, (B) Flu Near You, (C) athenahealth, and (D) HealthTweets.org for each influenza season.

## Abbreviations

CDC: Centers for Disease Control and Prevention
EHR: electronic health record
FNY: Flu Near You
ILI: influenza-like illness
ILINet: Influenza-like Illness Surveillance Network
IQR: interquartile range
SIR: susceptible-infected-recovered
