# Supplementary figures and images for: Differences in Regional Patterns of Influenza Activity Across Surveillance Systems in the United States: Comparative Evaluation
Source: JMIR Public Health Surveill. 2019 Sep 14;5(4):e13403. doi: 10.2196/13403 (PMC6777281; doi:10.2196/13403)

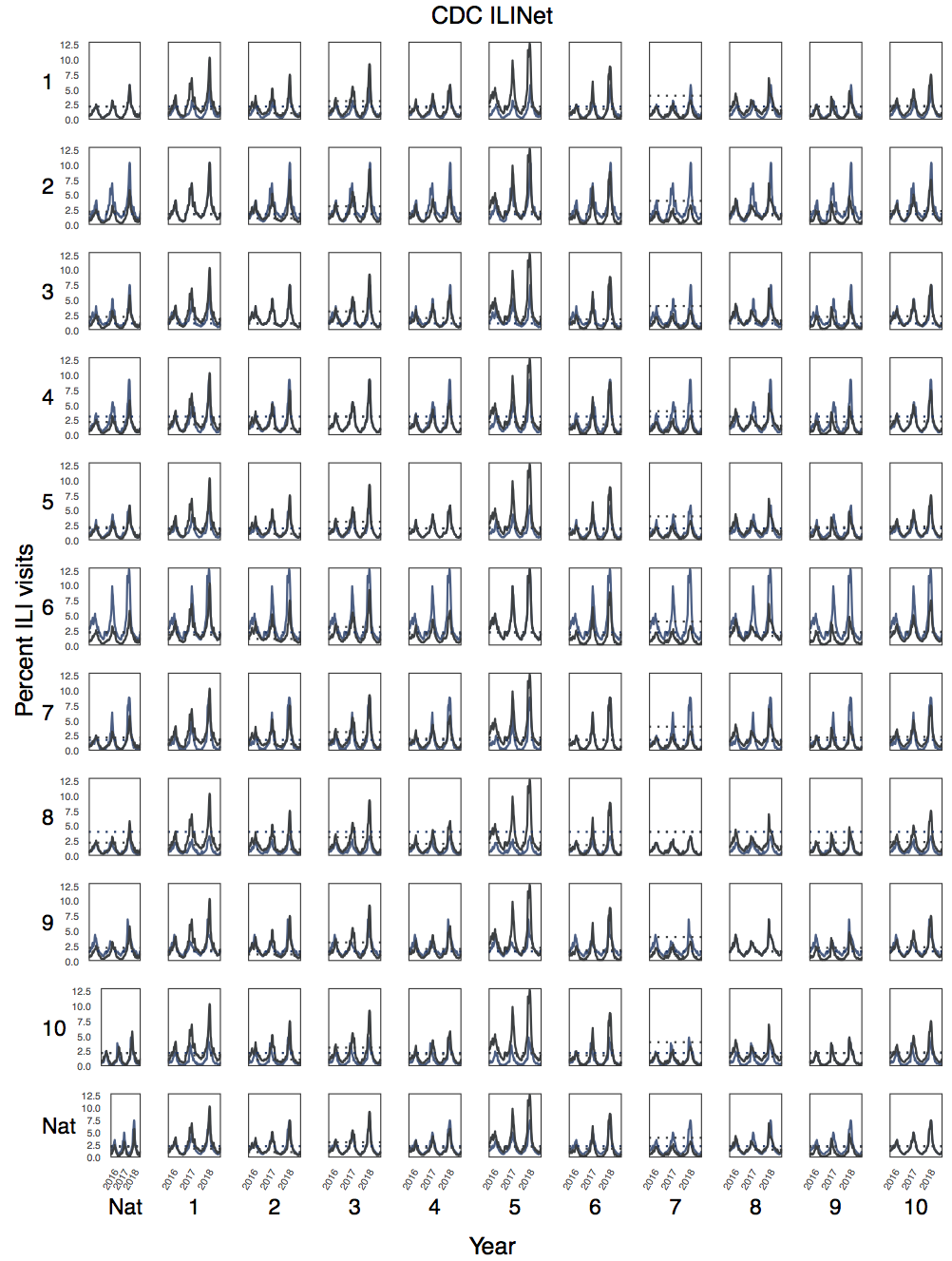

Supplement: Multimedia Appendix 1 [file publichealth_v5i4e13403_app1.png]

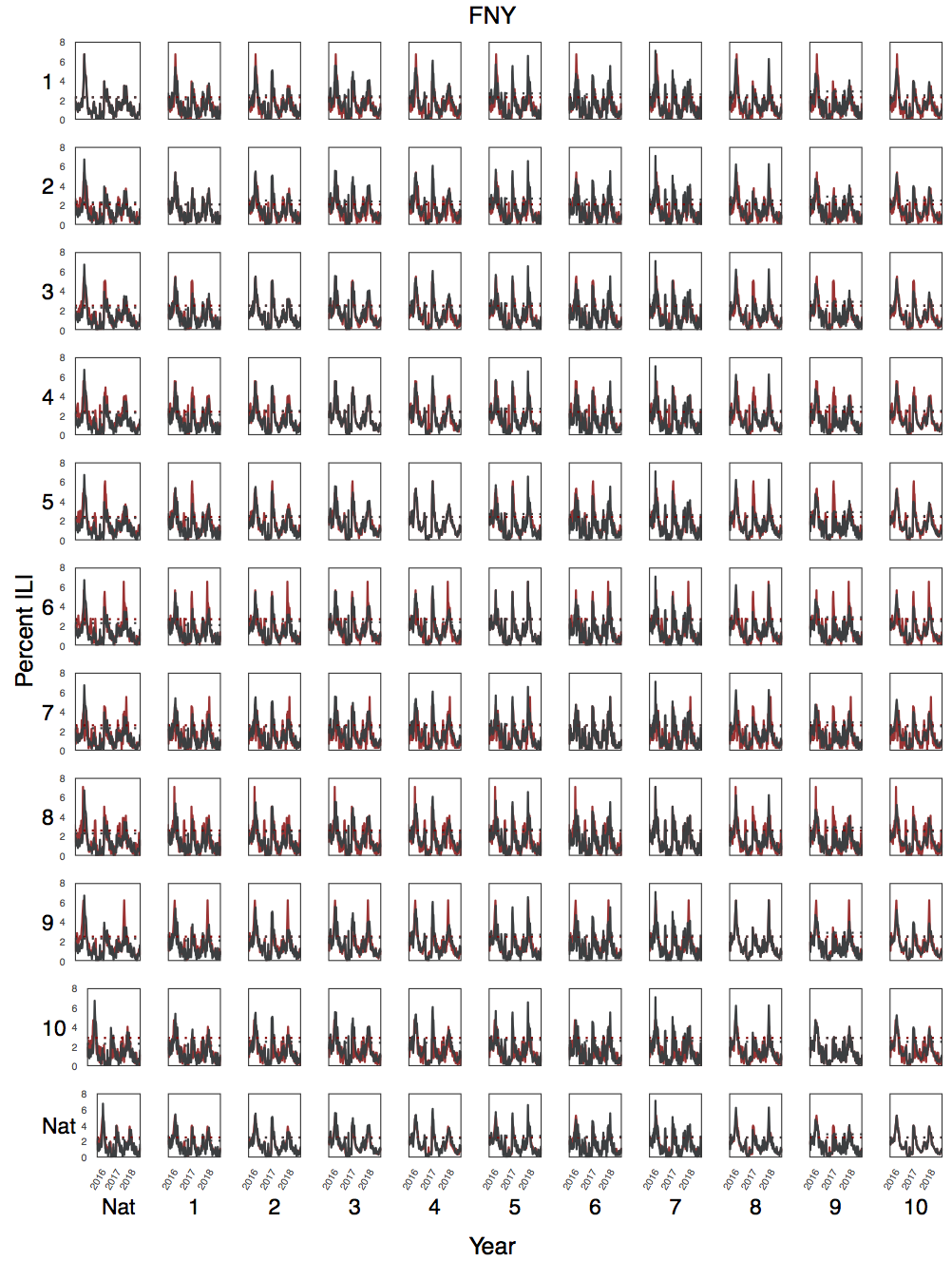

Supplement: Multimedia Appendix 2 [file publichealth_v5i4e13403_app2.png]

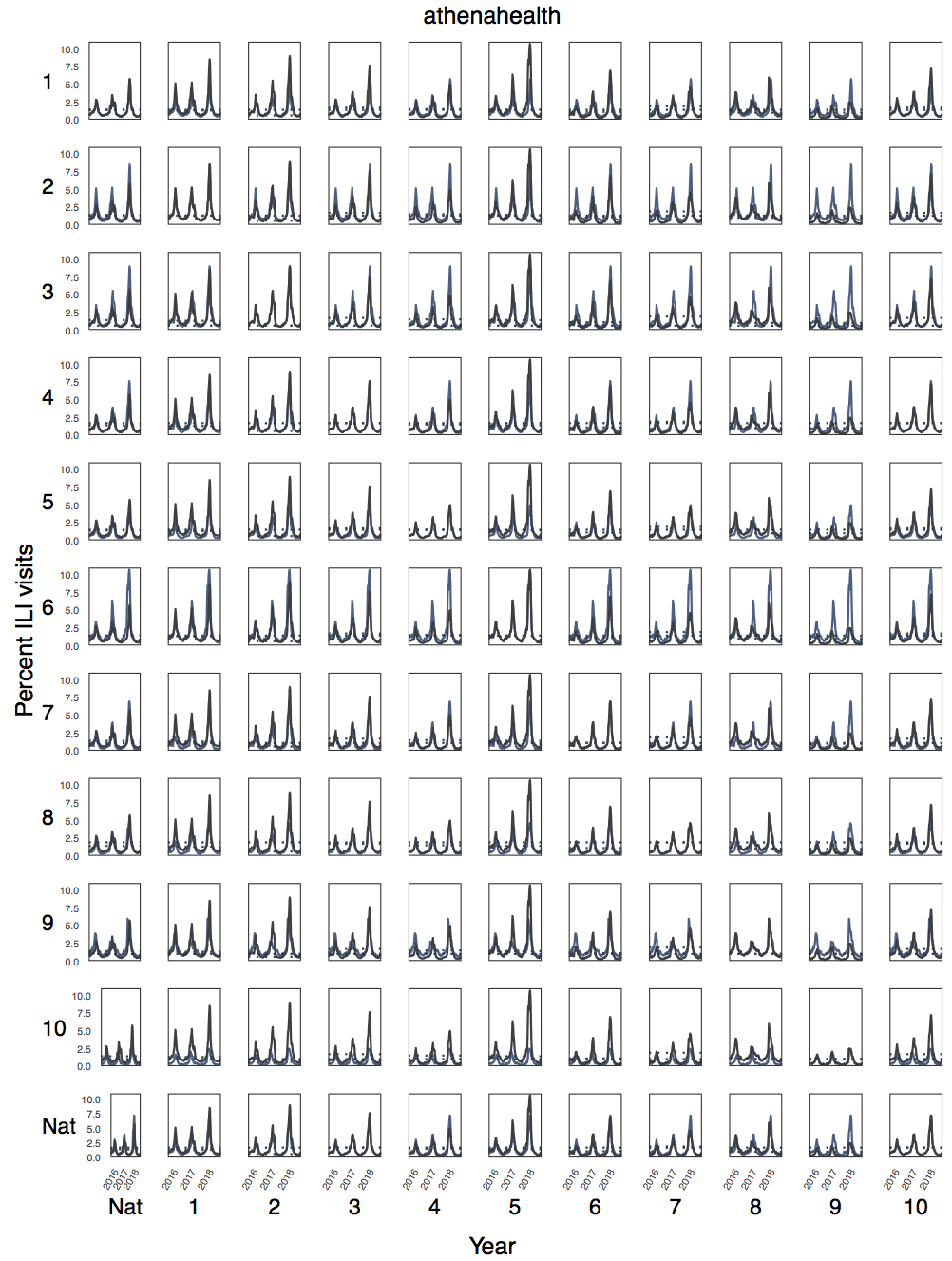

Supplement: Multimedia Appendix 3 [file publichealth_v5i4e13403_app3.png]

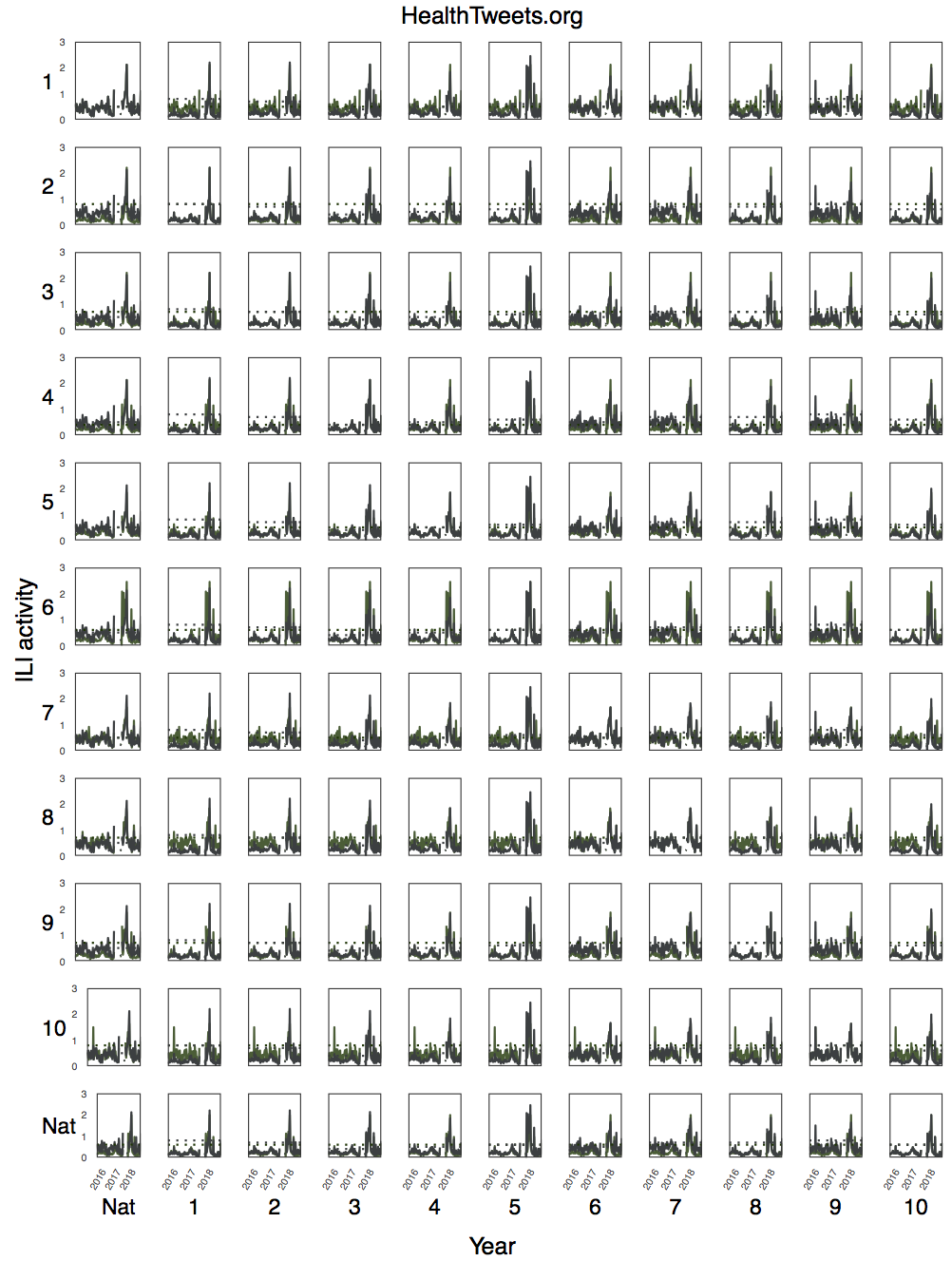

Supplement: Multimedia Appendix 4 [file publichealth_v5i4e13403_app4.png]

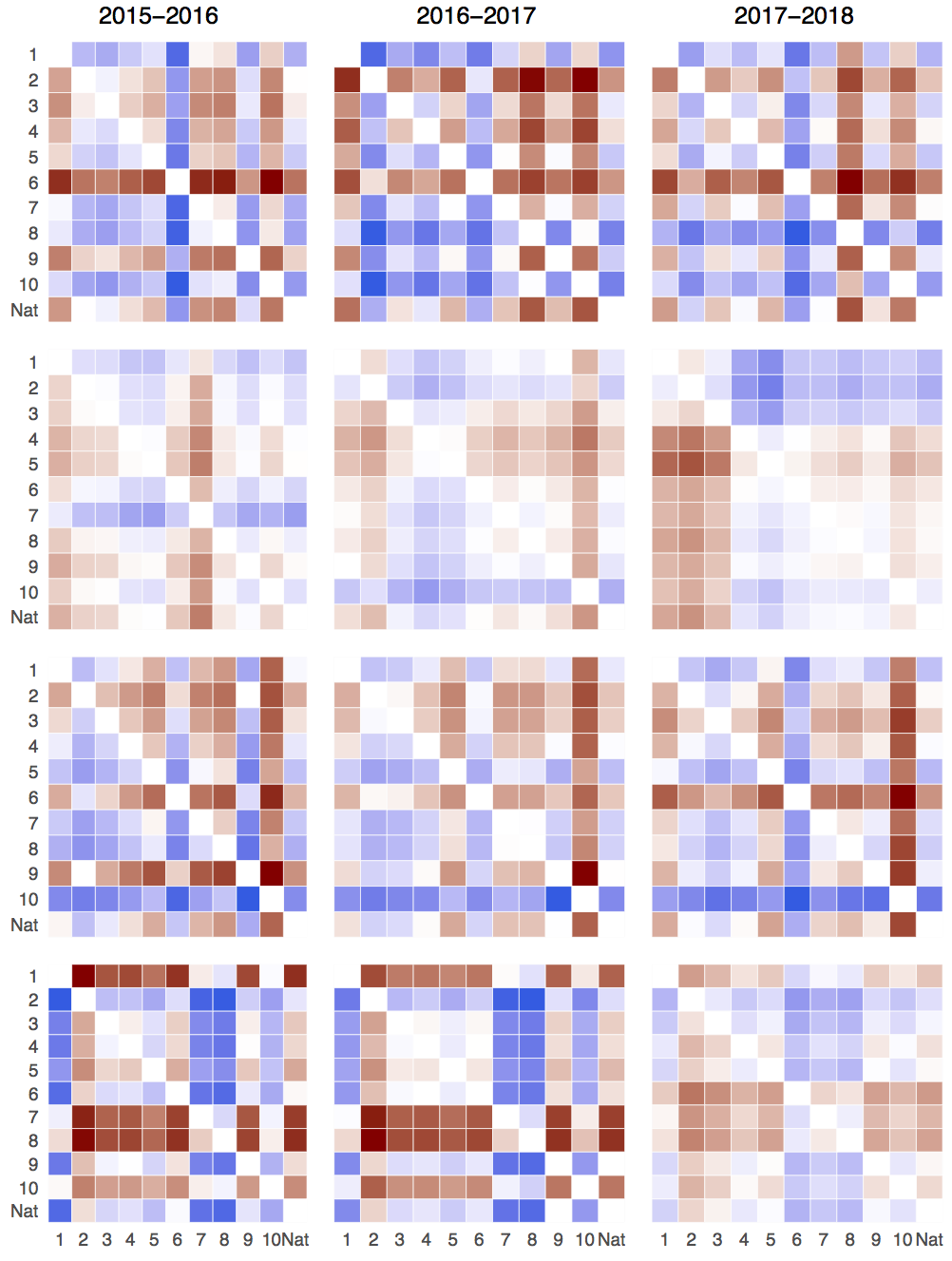

Supplement: Multimedia Appendix 5 [file publichealth_v5i4e13403_app5.png]
